# Designing an eco-efficient sonocatalytic process: iron-modified bamboo biochar for cyanobacteria elimination and algal organic matter valorization potential^☆^

**DOI:** 10.1016/j.ultsonch.2026.107988

**Published:** 2026-08-01

**Authors:** Yunxiao Zhu, Fulong Chen, Xiaoqing Qian, Juanjuan Wang, Xiaoge Wu, Juan Tu, Jun Wu

**Affiliations:** a College of Environmental Science and Engineering, Yangzhou University, Yangzhou 225009, China; b Key Laboratory of Cultivated Land Quality Monitoring and Evaluation, Ministry of Agriculture and Rural Affairs, Yangzhou 225009, China; c Key Laboratory of Modern Acoustics, Department of Physics, Collaborative Innovation Center of Advanced Microstructure, Nanjing University, Nanjing 210093, China; d Department of Geriatric Cardiology, Jiangsu Provincial Key Laboratory of Geriatrics, The First Affiliated Hospital of Nanjing Medical University, 300 Guangzhou Rd, Nanjing 210029, China

**Keywords:** Biochar, Ultrasound, Cyanobacteria removal, Sonocatalysis, Reactive oxygen species

## Abstract

Cyanobacterial blooms have emerged as a persistent threat to global aquatic ecosystem stability and drinking water safety. Conventional algae removal technologies (e.g., coagulation, photocatalysis) generally face two long-standing bottlenecks: the trade-off between removal efficiency and secondary pollution from cell lysis-triggered microcystin release, and the lack of valorization pathways for hazardous algal residues. In this study, a low-cost iron/potassium hydroxide co-modified bamboo biochar (BC-900-2, prepared via one-step pyrolysis at 900 °C with a Fe/KOH molar ratio of 2:1) was developed as a sonosensitizer to construct a synergistic sonocatalytic algae removal system. Under optimal conditions (400 mg/L dosage, 40 kHz ultrasound, 10 min), the integrated sonocatalytic process achieved over 90% inactivation of *Microcystis aeruginosa* (initial concentration: 2 × 10^6^ cells/mL). Critically, the residual dissolved microcystin-LR concentration was reduced from an initial 0.00105 mg L^−1^ to < 0.0002 mg L^−1^, thereby falling below the Chinese drinking water standard (0.001 mg L^−1^, GB 5749–2022). However, further validation at higher cyanobacterial densities and in natural water matrices is required. Mechanistic investigations revealed that the physical–chemical coupling yielded a synergy index of 1.82, originating from enhanced cavitation and sustained Fe^2+^/Fe^3+^ cycling, with hydroxyl radical (•OH) identified as the dominant reactive oxygen species responsible for algal cell inactivation and microcystin degradation. Beyond pollutant removal, molecular characterization by Fourier transform ion cyclotron resonance mass spectrometry (FT-ICR MS) indicated that the treated products were enriched in protein/amino sugar- and lignin-like components with moderate oxidation states, consistent with molecular signatures often associated with bioavailable organic matter. Pot experiments demonstrated that the treated algal products significantly increased soybean sprout root length and total length by 34% and 31%, respectively, supporting the feasibility of converting hazardous algal waste into a potential plant growth-promoting resource. This work proposes an integrated treatment-and-valorization paradigm for cyanobacterial bloom management that simultaneously addresses secondary pollution control and waste disposal. It should be noted that the current study was conducted at the laboratory scale; pilot-scale validation in real eutrophic water bodies is required to evaluate its practical applicability.

## Highlights

• Iron-modified bamboo biochar achieves >90% M. aeruginosa removal within 10 min of ultrasonication.
• Dissolved microcystin-LR concentration in effluent meets Chinese drinking water standard.
• Algal degradation products exhibit molecular features associated with bioavailability.
• Life cycle assessment confirms 73% lower operational energy consumption than single ultrasound process.
• The system realizes simultaneous cyanobacteria elimination and algal waste valorization for circular agriculture.

## Graphical abstract

**Figure:**
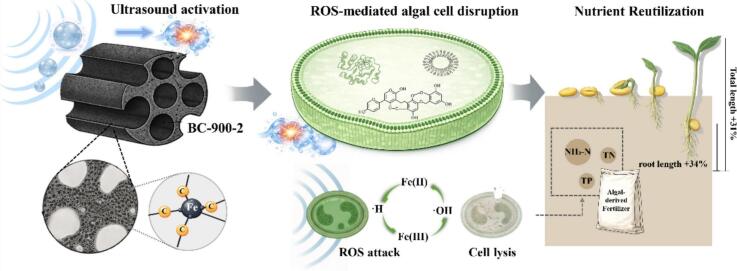

## 1 Introduction

The frequent proliferation of cyanobacterial blooms, driven by global water eutrophication and climate change, has become a persistent and escalating threat to aquatic ecosystem stability and drinking water safety worldwide. It is estimated that over 40% of global inland waters are affected by toxic cyanobacterial outbreaks annually, causing direct economic losses of more than $5 billion in the water sector alone [1], [2]. *Microcystis aeruginosa*, the dominant bloom-forming species, not only causes aesthetic deterioration, and ecological imbalance but also produces potent hepatotoxins (e.g., microcystins) and releases algal organic matter (AOM) during growth and lysis [3], [4]. These co-existing risks, including intact cells, intracellular toxins, and dissolved AOM, create a complex challenge for water treatment. Conventional algae management strategies, such as peroxidation − coagulation − flocculation, face limitations in removing dissolved toxins and AOM, and can even exacerbate the problem by causing cell lysis and subsequent toxin release [4], [5], [6]. Therefore, developing a technology that realizes simultaneous efficient algal cell inactivation, toxin degradation, AOM risk control, and algal biomass valorization remains a key challenge in cyanobacterial bloom management.

Ultrasound (US) technology has emerged as a promising physical–chemical approach for cyanobacterial control. Low-frequency ultrasound inactivates algae primarily through acoustic cavitation, which generates localized extreme condition, shear forces, and reactive oxygen species (ROS) like hydroxyl radicals (•OH) [7], [8]. Compared to chemical methods, US offers the advantage of no residual chemical addition and is less impeded by water turbidity. However, the practical application of single US treatment is limited by low energy transfer efficiency and insufficient ROS yield, which usually requires long treatment duration (>30 min) or high power input, resulting in high operation cost [9], [10].

Coupling US with functional materials as sonosensitizers is an effective strategy to solve the above bottlenecks. In the present study, BC-900-2 is described as a sonosensitizer because its function is specifically coupled to ultrasonic irradiation, and no external H_2_O_2_ was added during the algal treatment experiments. The porous biochar provides a solid–liquid interface for ultrasound-induced cavitation and facilitates the generation and transformation of oxidizing species during sonication. Compared with conventional sonosensitizers such as TiO_2_ and noble metal-based materials, the sonosensitizing mechanism of carbon-based materials is recognized to stem from their unique structural properties, including porosity, high conductivity, and tunable active sites. These properties synergistically enhance cavitation, promote charge separation, and mediate catalysis, thereby efficiently converting ultrasonic energy into reactive oxygen species (ROS) through multiple cooperative pathways [11], [12]. Biochar, a carbon-rich material derived from biomass pyrolysis, is an ideal candidate due to its tunable porosity, rich surface functional groups, and environmental compatibility, with a much lower production cost of commercial TiO_2_ sonosensitizers [13], [14].

When modified, biochar can transcend its traditional role as an adsorbent. For instance, KOH activation significantly increases its surface area and pore volume, improving interfacial contact and mass transfer [15], [16]. Loading iron species onto biochar introduces catalytic sites capable of activating H_2_O_2_ generated in situ during sonication through heterogeneous Fenton-like reactions, thereby amplifying ROS production [17], [18]. The stable Fe-O-C covalent bond formed between iron species and the carbon matrix can extend the pH adaptation range of the Fenton-like reaction to 3–10, covering the pH range of most natural water bodies [19], [20]. The synergistic effect of KOH-induced porous structure and Fe active sites is expected to greatly improve the sonocatalytic algae removal performance of biochar, with economic feasibility.

Despite the individual promise of US and modified biochar, their synergistic application for cyanobacterial control remains an under-explored frontier, particularly regarding the mechanistic difference between conventional TiO_2_-based composites and Fe-modified biochar for biomass valorization. While our prior work and others have demonstrated that TiO_2_/biochar composites can achieve algae removal coupled with resource recovery [16], the underlying mechanism has often been simplified as non-selective bulk oxidation. In those systems, although the feasibility of algal biomass valorization has been empirically validated, the molecular pathways by which cavitation-generated •OH radicals transform algal-derived organic matter into bioavailable fractions remain unclear. Without mechanistic clarity on the degradation trajectory, the retention of nitrogen/carbon-rich bioactive components could not be precisely controlled, limiting the rational design of sonocatalytic systems. In this study, by tracking reactive oxygen species at the biochar interface and resolving molecular transformation rules via FT-ICR MS, we clarify the Fe-O-C interfacial modulation mechanism. This mechanism steers the partial depolymerization of algal biopolymers, enabling deliberate control over the formation of bioavailable fractions rather than relying on empirical trial-and-error. This shift from oxidative destruction to controlled catalytic depolymerization provides a predictable route for converting hazardous algal waste into plant-growth promoters, aligning with SDG 6 and SDG 2 [21], [22].

To bridge the above knowledge and technological gaps, this study proposes a treatment-resource integration paradigm for cyanobacterial bloom management. We prepared an Fe/KOH co-modified bamboo biochar (BC-900-2) and coupled it with 40 kHz low-frequency US to construct a synergistic sonocatalytic system. Following a logical progression from material screening and structure–activity analysis to mechanistic elucidation and product characterization, this work ultimately validates the agricultural applicability of the treated biomass. This structured framework distinguishes empirical results from theoretical discussion, ensuring the robustness of our conclusions regarding both algal control and resource recovery. The research was carried out with three core objectives: (1) To optimize the preparation parameters of modified biochar, and evaluate its algal removal performance and microcystin control efficiency under different working conditions; (2) To elucidate the sonocatalytic mechanism through characterizing the material structure, detecting reactive species, and analyzing the algal cell injury process; (3) To clarify the molecular transformation rule of algal organic matter during degradation, verify the biological safety of degradation products, and evaluate their plant growth promotion effect via pot experiments. This work not only develops a low-cost, high-efficiency algae control technology, but also provides a feasible strategy for algal biomass valorization, supporting the sustainable management of cyanobacterial blooms. This study is a laboratory-based fundamental research, and its practical application performance requires further pilot-scale verification.

## 2 Methods

### 2.1 Material fabrication

Raw bamboo charcoal was washed with deionized water for 3–5 times and dried at 70 ℃ to constant weight. The dried bamboo charcoal powder was added to Fe_2_(SO_4_)_3_ solutions of different concentrations (0, 1, 2, 4 mol/L) at a solid–liquid ratio of 1:10 (g/mL), sealed with plastic wrap, stirred hydrothermally at 80℃ for 2 h using a magnetic stirrer at 200 rpm, filtered and dried at 105 °C. Then the iron-loaded biochar was mixed with KOH at a mass ratio of 1:3 and ground thoroughly in an agate mortar to achieve intimate solid–solid contact, followed by calcination in a tube furnace under nitrogen atmosphere (purge flow rate 200 mL/min) at a heating rate of 5 ℃/min, maintained at target temperatures (400 ℃, 900 ℃) for 2 h. After natural cooling to room temperature, the product was washed with deionized water to neutral pH, dried at 70 ℃, ground and sieved through 0.15 mm sieve to obtain modified biochar materials. The preparation method was optimized based on previous protocols [23], [24], [25] by adjusting iron loading concentration and pyrolysis temperature to meet the requirement of sonocatalytic algae removal performance.

### 2.2 Material characterization

The morphology, pore structure, crystal structure, surface functional groups, and elemental valence states of the materials were characterized systematically. Scanning electron microscopy (SEM, Regulus-8100, Hitachi High-Technologies Corporation, Tokyo, Japan), transmission electron microscopy (TEM, FEI Tecnai G2 F30 S-TWIN 300 kV, Thermo Fisher Scientific, Hillsboro, USA) and high-resolution transmission electron microscopy (HRTEM; FEI Talos F200X, USA) were employed to observe the surface and internal structure of materials. The specific surface area and pore size distribution were determined by nitrogen physisorption (BET, Quadrasorb EVO, Anton Paar, Graz, Austria), with samples degassed at 200 °C for 6 h before test. X-ray diffraction (XRD, AXS D8 ADVANCE, Bruker AXS, Karlsruhe, Germany) was performed in the 2θ range of 10-80° with a step size of 0.02° and scanning speed of 5°/min. Raman spectroscopy (Renishaw in Via, Renishaw, Wotton-under-Edge, UK) was tested with a 532 nm excitation laser. Fourier-transform infrared spectroscopy (FTIR, Cary 5000, Agilent Technologies, Santa Clara, USA) was scanned in the wavenumber range of 4000–400 cm^−1^. X-ray photoelectron spectroscopy (XPS, ESCALAB 250Xi, Thermo Fisher Scientific, Waltham, USA) was calibrated with C 1s = 284.8 eV as the reference binding energy.

### 2.3 Algae cultivation

*Microcystis aeruginosa* (FACHB-905) was purchased from the Freshwater Algae Culture Collection of the Institute of Hydrobiology, Chinese Academy of Sciences, and cultured in BG11 medium at 25 °C, 2000 lx, 12 h light/12 h dark cycle. Algae in the logarithmic growth phase were collected, centrifuged, and resuspended in sterile pure water. Cell concentrations were determined by direct microscopic counting using a hemocytometer, a calibrated microscope counting chamber with a defined grid and known chamber volume, under a light microscope. and were adjusted to approximately 2 × 10^6^ cells mL^−1^ by dilution with sterile pure water. [26] This concentration is a typical concentration for moderate cyanobacterial blooms in natural waters [27], [28].

### 2.4 Sonodynamic algae removal experiment

Sonodynamic algal treatment experiments were conducted using an ultrasonic cleaning bath operated at 40 kHz with a nominal output power of 200 W (SB-200DTD, YMANLY, Nanjing, China). The ultrasonic bath had external dimensions of 265 × 165 × 220 mm and internal tank dimensions of 240 × 135 × 100 mm, with a nominal capacity of 3.2 L. The bath water depth was maintained at 80 mm.

For each experiment, 20 mL of algal suspension was placed in a 50 mL glass beaker. The beaker was fixed at the horizontal geometric center of the ultrasonic bath, corresponding to a cavitation-active region identified by foil erosion mapping, with its bottom positioned approximately 65 mm above the tank bottom. The bath water level was maintained at or above the sample liquid level. The same vessel position, water depth, and immersion conditions were maintained throughout all experiments.

The system temperature was maintained at 25 °C using circulating water. The local acoustic intensity at the sample position was determined via the calorimetric method prior to biological experiments. Briefly, the temperature increase of 20 mL of deionized water (serving as the acoustic medium) in the same 50 mL beaker was monitored under ultrasonic irradiation using a calibrated thermocouple. The acoustic intensity (I, W cm^−2^) was calculated from the initial linear slope of the temperature-versus-time curve according to the following equation:

$$I=\frac{mC_{p}}{A}∙\frac{\mathrm{dT}}{\mathrm{dt}}$$

where mis the mass of the water (g), cp is the specific heat capacity of water (4.18 J g^−1^ °C^−1^), **A**is the cross-sectional area of the beaker base (cm^2^), and dT/dtis the initial heating rate (°C s^−1^). The calculated intensity was 0.3 W cm^−2^. It is critical to note that this value represents the local energy dose deposited into the confined sample volume at the fixed experimental position, rather than the spatially averaged intensity of the entire ultrasonic bath.

Four treatment groups were included: untreated algal control (no ultrasound or material), BC-900-2 alone (400 mg L^−1^ BC-900-2 without ultrasound), ultrasound alone (40 kHz ultrasound for 10 min without material), and BC-900-2 combined with ultrasound (400 mg L^−1^ BC-900-2 and 40 kHz ultrasound for 10 min).

After ultrasonic treatment, the samples were left undisturbed for 30 min to allow settling. The supernatant was then collected 2 cm below the liquid surface for subsequent analyses. All experiments were performed in triplicate.

### 2.5 Analytical methods for algal removal, cell damage, and algal organic matter

#### 2.5.1 Algal removal and cell-damage analysis

Algal cell concentrations were determined using a hemocytometer, and the algal cell removal efficiency was calculated as follows:

$$Algal\;cell\;removal\;efficiency=\frac{N_{0}-N_{t}}{N_{0}}\times 100\%$$

where $N_{0}$ is the initial algal cell concentration before treatment, and $N_{t}$ is the algal cell concentration in the collected supernatant after treatment and settling.

Cell membrane integrity was analyzed by flow cytometry (BD LSR Fortessa, BD Biosciences, USA). Samples were stained with SYTO 9 and propidium iodide (PI) according to the manufacturer’s protocols. Cellular metabolic activity was assessed using fluorescein diacetate (FDA, 10 μg/mL, 15 min incubation), which is hydrolyzed by intracellular esterases in viable cells to yield fluorescent fluorescein (detected at FITC 530/20 nm). Intracellular ROS levels were measured using 2′,7′-dichlorodihydrofluorescein diacetate (H_2_DCFDA, 10 μmol/L, 20 min incubation), which is deacetylated intracellularly to DCFH and oxidized by ROS to fluorescent DCF (FITC 530/20 nm). Preliminary controls (untreated algae, no dye) confirmed that chlorophyll autofluorescence (peak ∼ 680 nm, far-red) did not overlap the detection windows used (SYTO 9/FDA/H_2_DCFDA: FITC 530/20 nm; PI: PE 585/20 nm), so no spectral interference was observed.

#### 2.5.2 Water-quality and algal organic matter analyses

Chemical oxygen demand (COD) in the collected supernatant was determined using prefilled COD reagent vials and a multiparameter water-quality analyzer (SG-JL600, Henan Suijing Environmental Protection Technology Co., Ltd., Henan, China) according to the manufacturer’s instructions. COD removal efficiency was calculated from the COD concentrations before and after treatment.

Dissolved microcystin-LR in the collected supernatant was determined using a Microcystin Plate Kit (Beacon Analytical Systems Inc., USA) according to the manufacturer’s instructions.

For dissolved organic carbon (DOC) and ultraviolet absorbance at 254 nm (UV_254_) analyses, the collected supernatants were filtered through 0.45 μm polytetrafluoroethylene membranes. DOC was measured using a total organic carbon analyzer (Shimadzu, Japan). Samples were acidified and purged to remove inorganic carbon, followed by high-temperature catalytic oxidation and infrared detection of the generated CO_2_. UV_254_ was measured at 254 nm using a UV-2450 UV–visible spectrophotometer (Shimadzu, Japan) and a 1 cm quartz cuvette, with deionized water as the blank. Each sample was measured in triplicate [29], [30], [31].

EPS was sequentially fractionated into soluble EPS (S-EPS), loosely bound EPS (LB-EPS), and tightly bound EPS (TB-EPS). A 25 mL sample was centrifuged at 4000 rpm for 5 min, and the supernatant was collected as S-EPS. The pellet was resuspended to the original volume with 0.05% (w/v) NaCl and centrifuged at 5000 rpm for 10 min to obtain LB-EPS. The remaining pellet was resuspended again in 0.05% (w/v) NaCl, heated at 60 °C for 30 min, and centrifuged at 5000 rpm for 15 min to obtain TB-EPS[32]. Polysaccharide concentrations in the S-EPS, LB-EPS, and TB-EPS fractions were determined using the phenol–sulfuric acid method.

### 2.6 ROS detection and electrochemical characterization

Reactive oxygen species (ROS) generation was evaluated using multiple complementary techniques tailored to capture both bulk oxidative activity and specific radical species, with all assays performed in triplicate to ensure reproducibility.

Luminol-enhanced SCL, rather than intrinsic sonoluminescence, was employed to quantify cavitation-derived oxidative species. An alkaline luminol working solution (containing 1mmol L^−1^ luminol and 10mmol L^−1^ potassium ferricyanide) was added to each reaction system. The blue emission from luminol oxidation was captured using a digital camera with fixed, identical exposure settings (ISO 100, f/2.8, 30 s exposure) across all samples. A 30% H_2_O_2_ solution served as a positive control to calibrate the system’s sensitivity to oxidative species.

Bulk oxidative activity was further quantified via the KI assay. A 0.1mol L^−1^ KI solution was subjected to sonication for 10 min, and the absorbance of the oxidized product (I_3_^−^) was measured at 352 nm using a UV–vis spectrophotometer. The equivalent H_2_O_2_ concentration was calculated using a pre-established standard curve of I_3_^−^ absorbance versus H_2_O_2_ concentration (R^2^ > 0.99).

Hydroxyl radicals (•OH) were detected using a Bruker EMXplus-10/12 EPR spectrometer (Germany) with 5,5-dimethyl-1-pyrroline N-oxide (DMPO, 50mmol L^−1^) as the spin-trapping agent. Spectra were acquired at room temperature with the following standardized parameters: center field 3510 G, sweep width 200 G, modulation amplitude 1 G, microwave power 20 mW, and scan time 60 s. Radical quenching experiments were conducted to distinguish contributions of specific ROS: systems were pre-incubated with 100mmol L^−1^ *tert*-butanol (a specific •OH quencher) or 10mmol L^−1^p-benzoquinone (a specific O_2_•^−^ quencher) for 5 min prior to sonication.

Two selective molecular probes were used to track dominant ROS generation, consistent with the bulk assay results: 1,3-diphenylisobenzofuran (DPBF, 100 μM final concentration) for singlet oxygen (^1^O_2_) detection, and methylene blue (MB, 200 μM final concentration) for •OH detection. Absorbance changes were recorded at 410 nm (DPBF, reflecting ^1^O_2_-mediated probe oxidation) and 664 nm (MB, reflecting •OH-mediated probe bleaching) at predefined intervals (0, 2, 4, 6, 8, and 10 min) using a UV–vis spectrophotometer (Cary 5000, Agilent, USA).

Electrochemical measurements were performed using a CHI660E electrochemical workstation (CH Instruments, Shanghai, China) in a standard three-electrode configuration: working electrode (material-coated carbon paper, geometric area 1 cm^2^), reference electrode (saturated calomel electrode, SCE), counter electrode (platinum plate), and electrolyte (0.1mol L^−1^Na_2_SO_4_). Cyclic voltammetry (CV) was conducted over a potential window of − 0.8 to 0.8 V vs. SCE at a scan rate of 0.02 V s^−1^ to evaluate capacitive behavior and redox activity. Linear sweep voltammetry (LSV) was performed at a scan rate of 0.005 V s^−1^ to quantify interfacial polarization current density. Current–time (i–t) measurements were conducted at a constant applied potential of 0 V vs. SCE, with 40 kHz ultrasound (200 W, matching the sonodynamic treatment parameters) periodically applied and removed every 30 s for 5 consecutive cycles, to assess the dynamic interfacial charge response of materials to acoustic perturbation. The electronic structures of the materials were further characterized by UV–vis diffuse reflectance spectroscopy (Cary 5000, Varian, USA) to determine optical band gap, and XPS valence-band spectroscopy to resolve valence band edge position.

### 2.7 Nitrogen and phosphorus release capacity of algal-biochar flocs

The nitrogen and phosphorus release capacity of the materials was determined by leaching experiment. Biochar samples BC, BC-900–0, and BC-900-2 were separately mixed and reacted with the algal solution. The initial concentration of the algal solution was 2 × 10^6^ cells/mL, and the dosage of each biochar was 400 mg/L [33].The mixtures were ultrasonicated for 10 min (40 kHz, 200 W, matching the sonodynamic treatment parameters in Section 2.4) and subsequently allowed to settle quiescently for 30 min (consistent with the phase separation protocol for algae removal assays). The supernatant was then removed, and the settled algal-biochar flocs were collected for the 24 h leaching experiment. After the soaking period, the supernatant was collected for subsequent water quality analysis. To minimize interference from particulates, the extracts were centrifuged or filtered through a 0.45 μm membrane prior to determination. The measurements of NH_3_-N, TN, and TP in the extracts were conducted following standard methods for water and wastewater analysis·NH_3_-N was determined using the Nessler's reagent spectrophotometric method. TN was measured by the alkaline potassium persulfate digestion-UV spectrophotometric method. TP was analyzed using the ammonium molybdate spectrophotometric method after potassium persulfate digestion. For each analysis, all measurements were conducted in triplicate. The mean value and standard deviation (SD) were calculated and reported. Blank controls and standard solutions were included for all indicators.

### 2.8 Pot experiments

Four control groups were set to eliminate the interference of other factors: CK1 (pure water control), CK2 (diluted untreated algal solution), CK3 (single ultrasound treated algal solution), CK4 (BC-900-2 leaching solution). The test solution was the supernatant after ultrasonic treatment. For each treatment, 20 uniformly selected soybean seeds, pre-soaked for 6 h, were placed on filter paper in a Petri dish and supplied with 10 mL of the corresponding test solution. All treatments were set up in triplicate. The dishes were maintained at 25 ± 1 °C under a 12-h light/12-h dark photoperiod (2000 lx) for 7 days. Moisture was maintained by daily supplementation of the solutions. After the cultivation period, the germination rate was recorded. The mean value for each replicate was calculated from the 20 seedlings, and the data from the triplicate dishes were then used to determine the final mean ± standard deviation for statistical analysis.

### 2.9 Life cycle assessment methodology

This study systematically evaluated the environmental impacts of using iron-loaded biochar (BC-900-2) coupled with an ultrasonic system to remove *Microcystis aeruginosa* by applying the Life Cycle Assessment (LCA) method, in accordance with the ISO 14040 and 14,044 international standards [34], [35]. The functional unit was defined as the treatment of 1 cubic meter of algae-laden water. All modeling and analyses were performed using SimaPro 9.0 software, with background data sourced from the Ecoinvent 3 database and foreground data supplemented and adjusted based on actual experimental parameters. The system boundary encompassed three main stages: first, the raw material preparation stage, covering the acquisition and pretreatment of biomass; then, the material production stage, involving the high-temperature pyrolysis for BC-900-2 synthesis and the iron-loading process; and finally, the ultrasonic treatment stage, which accounted for the electricity consumption during the algae treatment process. Each stage was modeled as an independent subsystem before being integrated into a complete life cycle framework for combined analysis. The environmental impact assessment was carried out using the ReCiPe 2016 Midpoint (H) method, which covers 16 impact categories including global warming potential (GWP), stratospheric ozone depletion (SOD), ionizing radiation (IRP), ozone formation (OFHH, OFTE), fine particulate matter formation (PMFP), terrestrial acidification (TAP), freshwater eutrophication (FEP), ecotoxicity (TETP, FETP), human toxicity (HCTP, HNCTP), land use (LU), resource consumption (MRS, FRS), and water consumption (WC). The results were normalized and presented using percentage stacked bar charts to compare the relative contribution of each life cycle stage to the different impact categories. Furthermore, to specifically analyze the effect of the synergistic mechanism on energy consumption, the ultrasonic treatment subsystem was examined separately through a comparative analysis of two scenarios: “Ultrasound only (US only)” and “BC-900-2 coupled with Ultrasound (BC-900-2 + US).”.

## 3 Results

### 3.1 Preparation and performance screening of modified biochar

**Fig. 1:**
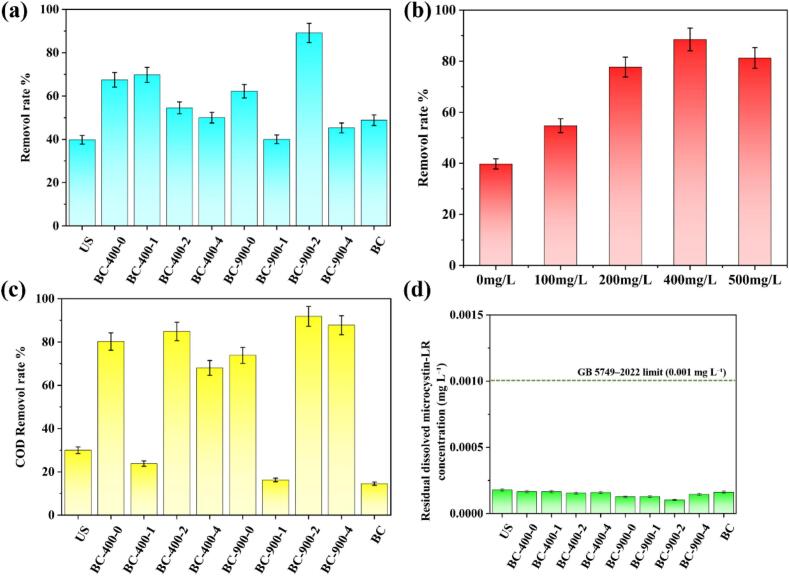
Sonodynamic performance of Fe/KOH co-modified biochar systems for algal removal and risk mitigation. (a) Algal cell removal efficiency of various modified biochar materials (coded according to Table S2) compared to raw biochar (BC) and ultrasound alone (US). The assays were conducted at a fixed dosage of 400 mg L^−1^ with 10 min of sonication followed by 30 min of quiescent settlement. (b) Dose-response relationship of BC-900-2 dosage (0–500 mg L^−1^) on algal removal efficiency. (c) Chemical oxygen demand (COD) removal efficiency corresponding to the treatments in panel (a). (d) Residual concentration of dissolved microcystin-LR (MC-LR) in the supernatant after treatment with the optimal BC-900-2/US system. The dashed horizontal line represents the maximum contaminant level (MCL) for MC-LR specified in the Chinese National Drinking Water Standard (GB 5749–2022, 0.001 mg L^−1^). The error bars represent the standard deviation (s.d.) of three independent replicates (n = 3).

Eight modified biochar samples were prepared using four Fe_2_(SO_4_)_3_ concentrations (0, 1, 2, and 4 mol L^−1^) and two pyrolysis temperatures (400 and 900 °C). Together with the unmodified raw bamboo biochar (BC), nine biochar samples were evaluated. The preparation conditions and nomenclature of all samples are summarized in Table S2. As shown in Fig. 1a, BC-900-2 exhibited the highest algal removal efficiency at a dosage of 400 mg L^−1^, reaching 91.2% ± 3.5% after 10 min of ultrasonic treatment followed by 30 min of quiescent settling.

Here, algal removal efficiency was operationally defined as the decrease in the concentration of suspended *Microcystis aeruginosa* cells in the collected supernatant relative to the initial cell concentration. Flow-cytometric analysis (Fig. S7a) revealed a pronounced increase in the membrane-compromised cell population in the BC-900-2/ultrasound system. Together with the EPR, radical-quenching, and intracellular ROS results, these findings support the contribution of ROS-mediated cellular injury to the observed reduction in suspended algal cells.

Nevertheless, gravitational settling and biochar-associated separation may also have contributed to the measured removal efficiency. The removal efficiency increased with increasing BC-900-2 dosage up to 400 mg L^−1^ but decreased slightly at 500 mg L^−1^ (Fig. 1b). This decrease may be associated with particle aggregation or acoustic shielding at the higher solid loading; however, these effects were not directly measured. Therefore, 400 mg L^−1^ was selected for subsequent experiments.

The COD removal efficiency was calculated based on the COD concentration of the supernatant collected 2 cm below the air–liquid interface after 10 min of combined BC-900-2/ultrasound treatment followed by 30 min of quiescent settling, relative to the initial algal suspension. This metric specifically evaluates the reduction of oxidizable organic matter remaining in the aqueous phase, excluding settled algal cells and biochar particles. The net COD reduction reflects the combined effects of intracellular AOM release from cell lysis, ROS-mediated oxidative degradation of dissolved organics, and adsorption of low-molecular-weight organics onto BC-900-2.

A BC-900-2-alone control (no ultrasound) showed no significant reduction in MC-LR compared to the initial concentration (0.00105 mg L^−1^), confirming that adsorption onto the biochar was negligible. In contrast, the combined BC-900-2/US treatment reduced the MC-LR concentration to below the Chinese drinking water standard (0.001 mg L^−1^). No detectable increase in dissolved MC-LR was observed under the tested laboratory conditions. However, this observation should be interpreted with caution, as it is specific to the current setup (2 × 10^6^ cells/mL, initial MC-LR 0.00105 mg L^−1^, pure-water matrix); higher cyanobacterial bloom densities, other microcystin congeners (e.g., MC-RR, MC-YR), and natural organic matter may alter MC-LR release and removal behavior, e.g., by competing for ROS and biochar adsorption sites. While the observed MC-LR removal may reflect both adsorption and ROS-mediated degradation [16], [29], [30], [31], the present experimental design limits independent quantification of these two pathways. Consequently, their relative contributions remain uncertain.

Pseudo-first-order kinetic modeling was applied to the decrease in suspended algal cell concentration over the 10 min treatment period. The apparent rate constant (k*_obs_*) for the BC-900-2/US system was 0.4213 min^−1^, which was approximately five times higher than that of the ultrasound-only control (0.085 min^−1^). This k*_obs_* represents the net rate of suspended cell removal, integrating both sonocatalytic inactivation and physical separation processes. Compared with other enhanced algae removal technologies (Table S1), this system has the advantages of short treatment time, no additional chemical agents, and simultaneous realization of algae removal and organic matter conversion, showing technical advantage. The algal concentration of 2 × 10^6^ cells/mL used in this study corresponds to the moderate outbreak level of cyanobacterial blooms in eutrophic lakes, which is typical representative. Natural water components such as humic acid may compete for active sites and reduce the treatment efficiency, and subsequent in-situ pilot tests will be carried out to verify the performance stability under complex water matrix conditions.

### 3.2 Structural characterization and structure–activity relationship of BC-900-2

**Fig. 2:**
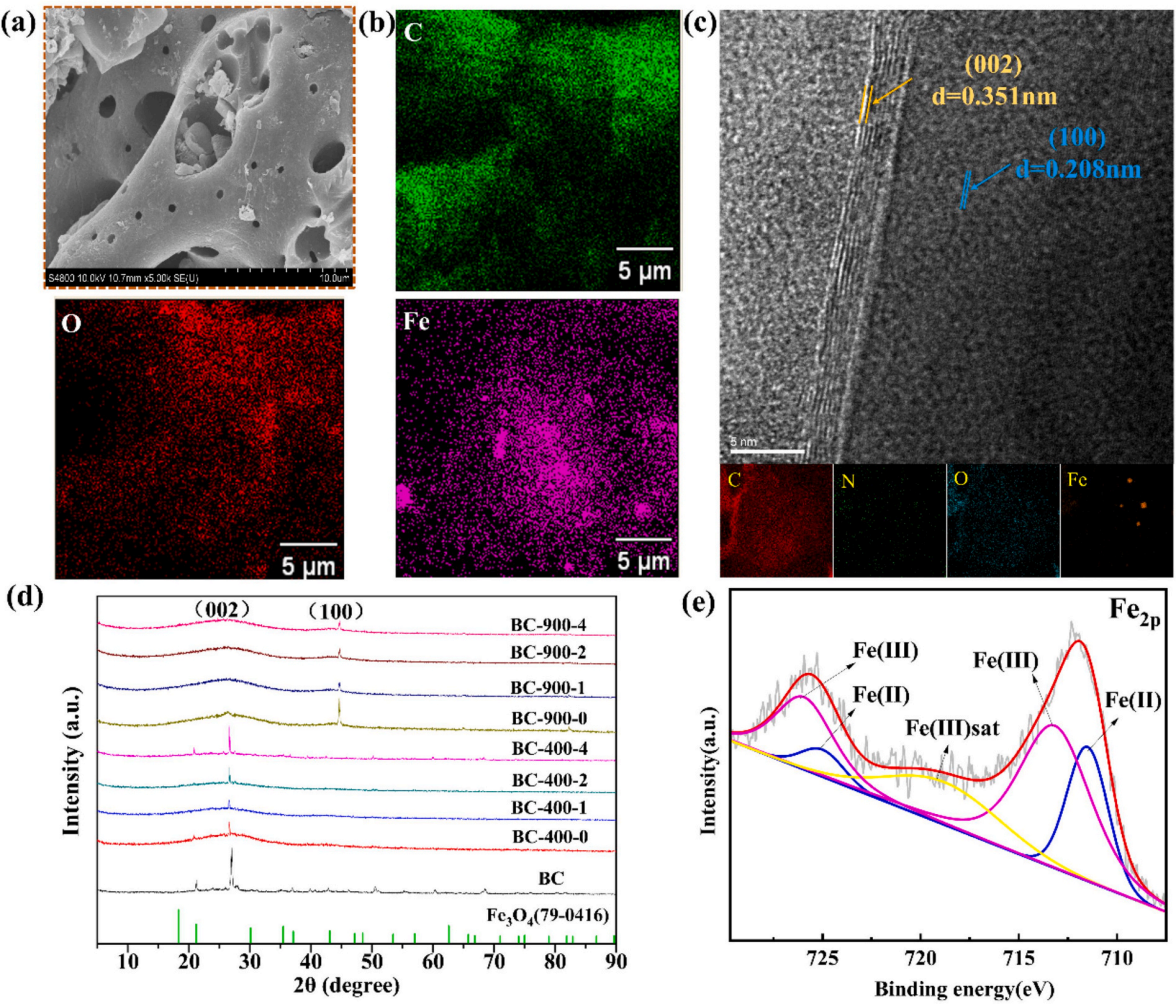
Multi-scale structural characterization of the optimal sonosensitizer BC-900-2 (a) Scanning electron microscopy (SEM) image of BC-900-2, scale bar = 1 μm. (b) SEM-energy dispersive spectroscopy (EDS) elemental mapping showing the homogeneous distribution of C, O, and Fe throughout the BC-900-2 matrix. (c) High-resolution transmission electron microscopy (HRTEM) image of BC-900-2, the lattice fringes of 0.351 nm and 0.208 nm correspond to the (002) plane of graphitic carbon and (1 0 0) plane of Fe_3_O_4_, respectively, scale bar = 5 nm. (d) X-ray diffraction (XRD) patterns of BC-900-2 and control samples, confirming the coexistence of graphitic carbon and crystalline Fe_3_O_4_ (JCPDS No. 79–0416). (e) X-ray photoelectron spectroscopy (XPS) Fe 2p high-resolution spectrum of BC-900-2, confirming the coexistence of Fe^2+^ and Fe^3+^.

Multi-scale characterization was carried out to clarify the structural basis for the high sonosensitization activity of BC-900-2, and the structure-performance correlation was established by comparing with raw BC and iron-free modified BC-900-0. SEM images showed that BC-900-2 had uniform pore distribution, rough surface and no obvious Fe particle agglomeration, and SEM-EDS mapping confirmed that Fe was uniformly dispersed in the carbon matrix (Fig. 2) [36]. BET analysis showed that BC-900-2 had a specific surface area of 925.774 m^2^ g^−1^, a total pore volume of 0.138 cm^3^ g^−1^, and an average pore diameter of 5.002 nm. The pore-size distribution, derived from the Barrett-Joyner-Halenda model, was predominantly within the mesoporous range (2–50 nm), exhibiting a major peak at approximately 3–4 nm (Fig. S2.) [37], [38], [39].This mesoporous architecture did not permit the entry of micrometer-sized algal cells; rather, it increased the accessible external interfacial area for algal-cell adhesion and provided short-range diffusion pathways for dissolved reactive species (e.g., •OH) and algal organic matter toward the Fe-O-C active sites.

Raman spectra exhibited characteristic D and G bands at approximately 1350 and 1580 cm^−1^, respectively. The D band is associated with defects and structural disorder in the sp^2^ carbon network, whereas the G band corresponds to the in-plane vibration of graphitic sp^2^ carbon; therefore, the intensity ratio I_D_/I_G_ is commonly used to evaluate the relative defect density and structural disorder of carbon materials. BC-900-2 exhibited a higher I_D_/I_G_ ratio (1.039) than raw BC (0.722), indicating a greater abundance of defect and edge sites that could provide additional interfacial active sites and facilitate electron transfer during sonocatalytic reactions [40]. FTIR and XPS results confirmed the existence of Fe-O-C bond in BC-900-2, and Fe existed in the mixed valence state of Fe^2+^/Fe^3+^, which provided the basis for Fenton-like cycle [41].

High-resolution transmission electron microscopy (HRTEM) imaging (Fig. 2c) revealed local graphitized domains in BC-900-2, with lattice spacings of 0.351 and 0.208 nm assigned to the (002) plane of graphitic carbon and the (1 0 0) plane of Fe_3_O_4_, respectively [42]. The corresponding elemental mappings further indicated the dispersed distribution of Fe-containing species. The above characterization results confirmed that BC-900-2 had the structural advantages of high specific surface area for algal adsorption, uniformly dispersed Fe active sites for Fenton-like reaction, stable Fe-O-C bonding for electron transfer, and local graphitized carbon skeleton for cavitation enhancement, which collectively contributed to its high sonocatalytic activity.

The parameter selection of this study refers to the optimal preparation range of reported iron-modified biochar catalytic materials: when the pyrolysis temperature is lower than 700 ℃, it is difficult for iron species to form stable Fe_3_O_4_ crystal phase and Fe-O-C bonding structure, while temperatures higher than 1000℃ will lead to excessive graphitization of the carbon skeleton and pore collapse. Therefore, two typical temperatures of 400℃ (low-temperature control) and 900℃ (optimal activity interval) were selected; the Fe loading concentration refers to the conventional iron loading gradient of Fenton-like catalysts, and the 2 mol/L loading can achieve the optimal balance between active site dispersion and catalytic activity, while higher concentrations will lead to iron particle agglomeration; the mass ratio of KOH to biochar of 1:3 is a classic ratio for bamboo-based biochar activation, which can achieve the optimal regulation of specific surface area and pore structure.

### 3.3 Sonodynamic algae removal mechanism

To elucidate the sonodynamic algae removal mechanism of BC-900-2, we conducted multi-method experiments to verify a triple synergistic mechanism: enhanced cavitation, interfacial electron transfer with sustained Fe^2+^/Fe^3+^ cycling, and ROS-mediated cellular damage. The experimental evidence is organized sequentially below.

#### 3.3.1 Cavitation enhancement and ROS generation

**Fig. 3:**
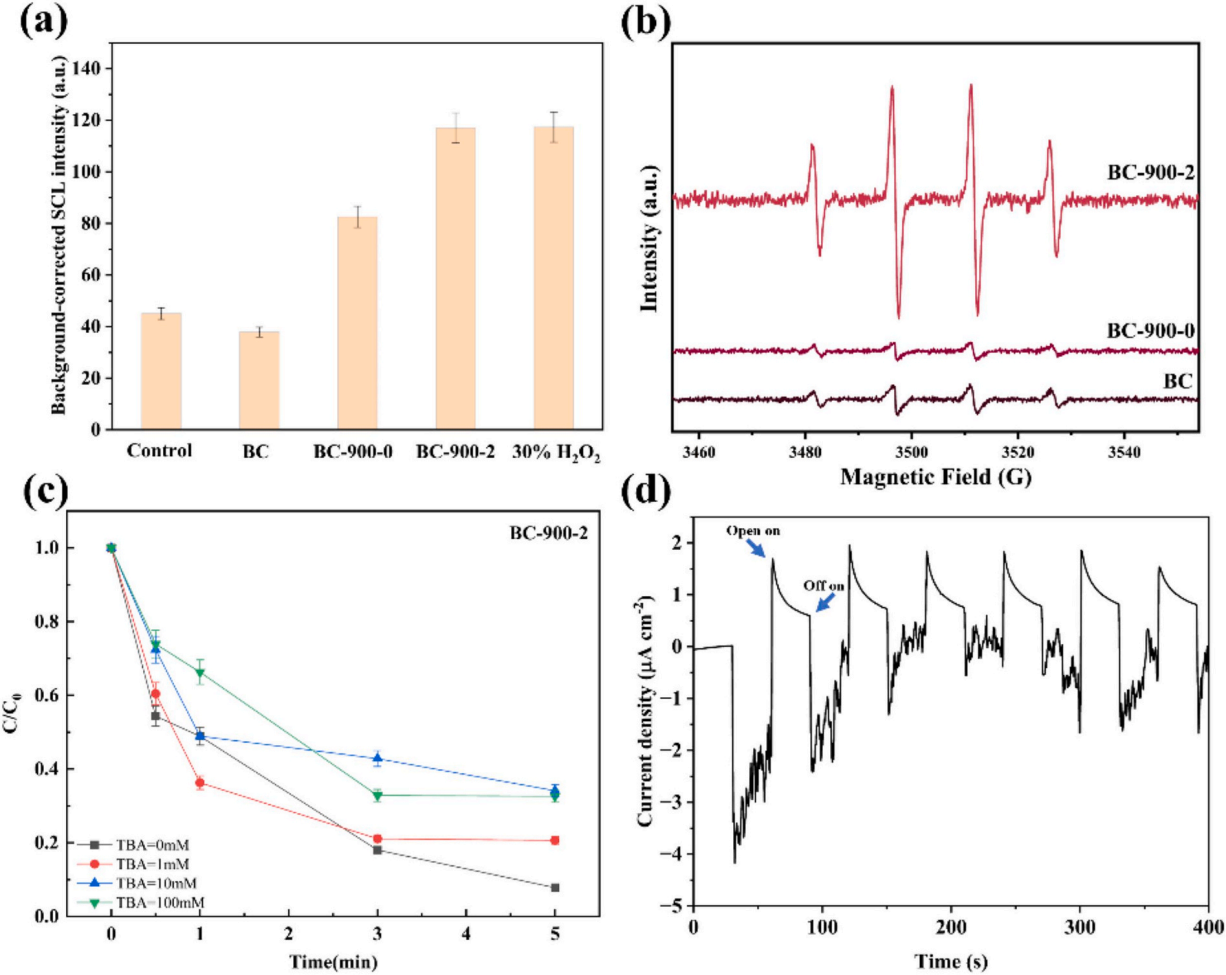
Elucidation of the sonodynamic activation mechanism of BC-900-2. (a) ImageJ-based quantification of luminol-enhanced sonochemiluminescence (SCL) intensity. SCL intensity was calculated from blue-channel grayscale images using the same region of interest (ROI) after background subtraction. (b) Electron paramagnetic resonance (EPR) spectra of DMPO–•OH adducts in different systems. (c) Algal degradation kinetics (C/C_0_ over time) in the BC-900-2/US system under different concentrations of *tert*-butanol (TBA). (d) Current–time response of BC-900-2 under periodic ultrasound on/off conditions, inset shows stability over 5 consecutive cycles.

Luminol-enhanced sonochemiluminescence (SCL) assays were used to evaluate cavitation-related oxidative activity in different systems. ImageJ-based blue-channel grayscale analysis showed that the background-corrected SCL intensity of the BC-900-2/US system was 3.09 and 1.42 times those of the raw BC/US and BC-900–0/US systems, respectively, and was comparable to that of the 2 mL 30% H_2_O_2_ positive control (Fig. 3a, Fig. S4) [43], [44], [45], indicating that BC-900-2 significantly promoted the generation of oxidative active species under ultrasonic field. To ensure the SCL intensity accurately reflected chemical activity rather than optical artifacts (e.g., light scattering or absorption by biochar particles), we implemented a rigorous standardization protocol. All images were captured using identical camera settings (ISO, aperture, exposure time) and a fixed region of interest (ROI). The SCL intensity was background-subtracted using signals from biochar suspensions without luminol. Control experiments confirmed that the presence of biochar particles did not significantly quench the luminescence signal under the experimental settings.

KI oxidation tests further quantified the total oxidation activity of the system: the equivalent H_2_O_2_ production in BC-900-2/US system reached 78.2 μmol/L after 10 min treatment, which was 3.2 times that of the single US system. The oxidation activity increased gradually with temperature rising from 15 °C to 45 °C (Fig. S5). This positive temperature dependence differs from conventional sonochemical degradation, where elevated temperature typically suppresses cavitation efficiency by increasing the vapor pressure within bubbles, thereby shortening the hot-spot lifetime [46], [47]. In the BC-900-2/US system, however, the Fe–O–C interfacial sites sustain a Fenton-like cycle that activates in situ H_2_O_2_. The kinetics of this heterogeneous catalytic process accelerate significantly with temperature, outweighing the modest cavitation penalty and leading to the observed net increase in oxidative yield[48]. These complementary assays were designed with distinct, non-redundant roles: the KI oxidation assay quantified bulk oxidative yield (H_2_O_2_ equivalents) as a non-selective indicator; MB (•OH) and DPBF (^1^O_2_) molecular probes tracked relative ROS-specific trends; EPR spin trapping provided definitive spectroscopic identification of short-lived •OH (the 1:2:2:1 quartet); and TBA quenching quantified the functional contribution of •OH to the biological endpoint (algal removal), distinguishing mere generation from cytotoxic relevance.

Electron paramagnetic resonance (EPR) tests detected a typical 1:2:2:1 quartet characteristic signal of DMPO-•OH adduct in the BC-900-2/US system, with the signal intensity 4.1 times that of single US system and 2.6 times that of BC-900–0/US system, directly confirming the massive generation of •OH (Fig. 3b) [49]. After adding 100 mM *tert*-butanol (TBA, specific •OH quencher), the algae removal efficiency of BC-900-2/US system decreased from 91.2% to 29.2%, with a reduction of 68%, while the removal efficiency of single US system only decreased by 32%, verifying that •OH was the dominant reactive species in the system [50], [51] (Fig. 3c). Probe experiments showed that the absorbance of methylene blue (MB, •OH indicator) decreased by 82% within 10 min, while that of 1,3-diphenylisobenzofuran (DPBF, ^1^O_2_ indicator) decreased by 27% (Fig. S6), confirming that ^1^O_2_ acted as a side reactive species in the reaction [52]. In addition to the contribution of free radicals (∼95% by TBA quenching), the remaining removal efficiency may originate from the physical damage to algal cell gas vesicles and cell walls caused by mechanical shear forces and shock waves generated by ultrasonic cavitation.

#### 3.3.2 Interfacial electron transfer and Fe redox cycling

We next investigated how BC-900-2 sustains continuous ROS generation, focusing on its interfacial charge regulation ability. Electrochemical tests revealed the interfacial charge regulation ability of BC-900-2. The cyclic voltammetry (CV) curve showed no obvious redox peak in the potential window of −0.8 ∼ 0.8 V (vs SCE), indicating that its electrochemical behavior was dominated by electric double layer capacitance; the linear sweep voltammetry (LSV) curve showed that its interfacial polarization current density was 1.8 times that of raw BC, reflecting stronger charge accumulation capacity. The current–time (i-t) response curve showed that the current rapidly increased by 1.2 μA when ultrasound was turned on, and quickly returned to the baseline after ultrasound was turned off, with no obvious attenuation after 5 cycles, confirming the rapid and reversible interfacial charge response ability of BC-900-2 to ultrasonic perturbation (Fig. 3d) [53], [54].Band-structure analysis was performed to establish the thermodynamic baseline for redox reactions at the BC-900-2/water interface. The apparent optical band gap (E_g_) of BC-900-2 was 1.74 eV, derived from UV–vis diffuse-reflectance spectroscopy (Fig. S6). Crucially, the valence-band (VB) edge position was directly determined by XPS valence-band spectroscopy (Fig. S6), yielding a VB edge of 2.28 eV vs. NHE (Fig. S6). The corresponding conduction-band (CB) edge is estimated as ∼0.54 eV vs. NHE (E_cb_ = E_vb_−E_g_). This XPS-DRS combination is a standard protocol for carbon-based composites [55], [56]. The VB edge (+2.28 eV) is sufficiently positive to thermodynamically permit •OH generation from adsorbed H_2_O or OH^−^ (E^∘^(∙OH/H_2_O) = 2.38 V vs. NHE). While cavitation generates photons (>3.5 eV), the primary role of the band structure is to define thermodynamic redox potentials available at the Fe–O–C interface. Charge separation is attributed to ultrasound-driven piezoelectric or thermal effects at the locally graphitized carbon skeleton and Fe_3_O_4_ sites, as evidenced by the rapid i–t response (Fig. 3d), rather than direct photoexcitation.

To verify that this rapid charge response facilitates Fe redox cycling, X-ray photoelectron spectroscopy (XPS) and Fourier transform infrared spectroscopy (FTIR) were employed to characterize the post-reaction interface (Fig. S3 and S8). XPS analysis revealed a significant increase in the proportion of O–C=O groups (from 8.2% to 13.7%) and Fe^3+^ (from 58.4% to 72.1%), accompanied by a corresponding decrease in Fe^2+^. This inversion in Fe valence states provides strong evidence for the ultrasound-driven Fe^2+^/Fe^3+^ redox cycling. Complementarily, FTIR spectra showed an enhanced Fe–O stretching vibration and diminished intensities of C–O and C=O bonds. These spectral shifts are indicative of interfacial interactions between the catalyst and algal organic matter (AOM). Specifically, the attenuation of C=O/C–O signals coupled with the emergence/enhancement of Fe–O signals suggests the formation of Fe–O–C linkages or surface complexes via ligand exchange between iron oxyhydroxide sites and carboxyl-rich AOM components [57], [58]. Consistent with previous studies [26], [57], these enriched surface oxygen moieties likely facilitate the adsorption and stabilization of protein-like and carboxyl-rich AOM degradation products, thereby integrating the catalytic redox cycle with the removal of dissolved organic pollutants. Collectively, these interfacial modifications, corroborated by EPR and electrochemical data, suggest the sustained ROS generation responsible for algal cell inactivation.

#### 3.3.3 Ros-mediated cellular damage and proposed mechanism

Having confirmed sustained ROS generation, we next examined whether these ROS caused damage to algal cells. Flow-cytometric analyses were used to evaluate membrane integrity, intracellular esterase activity, and intracellular ROS levels in *Microcystis aeruginosa* cells. SYTO 9/PI double staining showed that the BC-900-2/ultrasound treatment produced the largest increase in the membrane-compromised cell population compared with the untreated control, ultrasound alone, and BC-900-2 alone (Fig. S7a). FDA staining further showed a marked decrease in intracellular esterase activity, indicating reduced cellular metabolic activity and viability (Fig. S7b). In parallel, H_2_DCFDA fluorescence increased substantially after the combined treatment, demonstrating enhanced intracellular oxidative stress (Fig. S7c). It is important to note that these samples were collected immediately following the 10-min ultrasonic treatment and 30-min quiescent settling period. Due to the absence of time-resolved measurements in this study, these observations represent complementary endpoints of cellular injury rather than a defined temporal sequence of damage. The BC-900-2/US system achieved a 68% reduction in algal removal efficiency upon the addition of 100 mM TBA (Fig. 3c), confirming that •OH-mediated oxidative damage was the dominant mechanism for cell inactivation. While the contribution of free radicals was predominant (∼95% by TBA quenching), the residual removal efficiency (approximately 29.2%) and SEM observations of cell wall rupture (Fig. S7d) suggest that mechanical shear forces and shock waves generated by ultrasonic cavitation contributed to physical disruption. Rather than establishing a strict chronological order, we conclude that the synergistic action of physical disruption and sustained ROS generation leads to irreversible cell damage. The rapid current response of BC-900-2 under ultrasonic perturbation (Fig. 3d), imposed continuous oxidative pressure on the algal cells.

To quantitatively distinguish the cooperative effect from a mere additive contribution, a synergy index (SI*_UV254_*) was calculated based on the UV_254_ absorbance of transformed algal organic matter (Supplementary Text S1). The calculated SI*_UV254_* was 1.82, which is significantly greater than 1. This result provides quantitative evidence that the interaction between BC-900-2 and ultrasound is synergistic, producing a stronger transformation of UV-active organic components than the sum of individual treatments.

Based on the above experimental results, we proposed a triple synergetic sonodynamic mechanism for BC-900-2, which is fully supported by characterization data. The developed mesoporous structure of BC-900-2 provides abundant nucleation sites for cavitation bubbles, and the enhanced cavitation-related oxidative activity was supported by the ImageJ-quantified SCL intensity. The enhanced cavitation effect promotes the cleavage of water molecules to generate •OH and H_2_O_2_ under the instantaneous high-temperature and high-pressure conditions created by bubble collapse. The locally graphitized carbon skeleton of BC-900-2 improves interfacial electron conduction efficiency, which is verified by the 1.8 times higher interfacial polarization current density of BC-900-2 than raw biochar, accelerating the electron transfer process during the reaction. The uniformly dispersed Fe_3_O_4_ active sites on BC-900-2 catalyze the reaction between Fe^2+^ and in-situ generated H_2_O_2_ to generate additional •OH, forming a sustained Fenton-like cycle. The Fe^2+^/Fe^3+^ cycle is sustained under the combined action of ultrasound reduction and carbon skeleton-mediated electron transfer, which is confirmed by the 13.7% decrease of Fe^2+^ proportion after reaction and rapid current response under ultrasonic perturbation, enabling continuous ROS generation. This sustained ROS generation likely maintained oxidative pressure on the algal cells throughout sonication. Together with the EPR, radical-quenching, SYTO 9/PI, FDA, and H_2_DCFDA results, the data support ROS-mediated oxidative damage as a major contributor to membrane impairment, reduced metabolic activity, and intracellular oxidative stress in the BC-900-2/ultrasound system.

### 3.4 Characteristics of algal degradation products and resource utilization potential

Beyond high algae removal efficiency, we further systematically characterized the composition, structural features and agricultural valorization potential of algal organic matter (AOM) generated from the sonodynamic system, to explore the feasibility of transforming cyanobacteria pollutants into bioavailable resources.

Dissolved organic carbon (DOC) measurement showed distinct release patterns of AOM across different treatment systems. The untreated raw algae (MA) group had the lowest DOC content as most organic matter was retained in intact algal cells. Single ultrasound (US) treatment increased DOC to 16.2 mg/L by inducing partial cell lysis. While the BC-900-2/US system yielded the highest DOC of 28.7 mg/L, which was 1.77 times that of the single US system, confirming the synergistic effect significantly promoted the release of intracellular organic matter from lysed cells. The UV_254_ value of the BC-900-2/US system reached 0.41, which was 1.86 times that of the MA group and 1.41 times that of the single US group, indicating that the released AOM had moderate aromaticity and conjugated structure, rather than being completely mineralized into small inorganic molecules (Fig. S9a-b).

Extracellular polymeric substance (EPS) analysis further revealed the disturbance effect of the system on algal cell surface structure. The total EPS polysaccharide content of the BC-900-2/US system reached 120.15 μg, which was 1.45 times that of the MA group (82.86 μg). Among different EPS fractions, the content of tightly bound EPS (TB-EPS) increased most significantly to 66.00 μg, which was 2.17 times that of the MA group, while soluble EPS (S-EPS) and loosely bound EPS (LB-EPS) showed no obvious fluctuation. This result may come from two synergistic processes. Ultrasonic effect strips the tightly bound organic layer on the algal cell surface, and intracellular organic components released by cell lysis are adsorbed by biochar and attached to the EPS fraction, the contribution ratio of which will be quantified in subsequent studies.

**Fig. 4:**
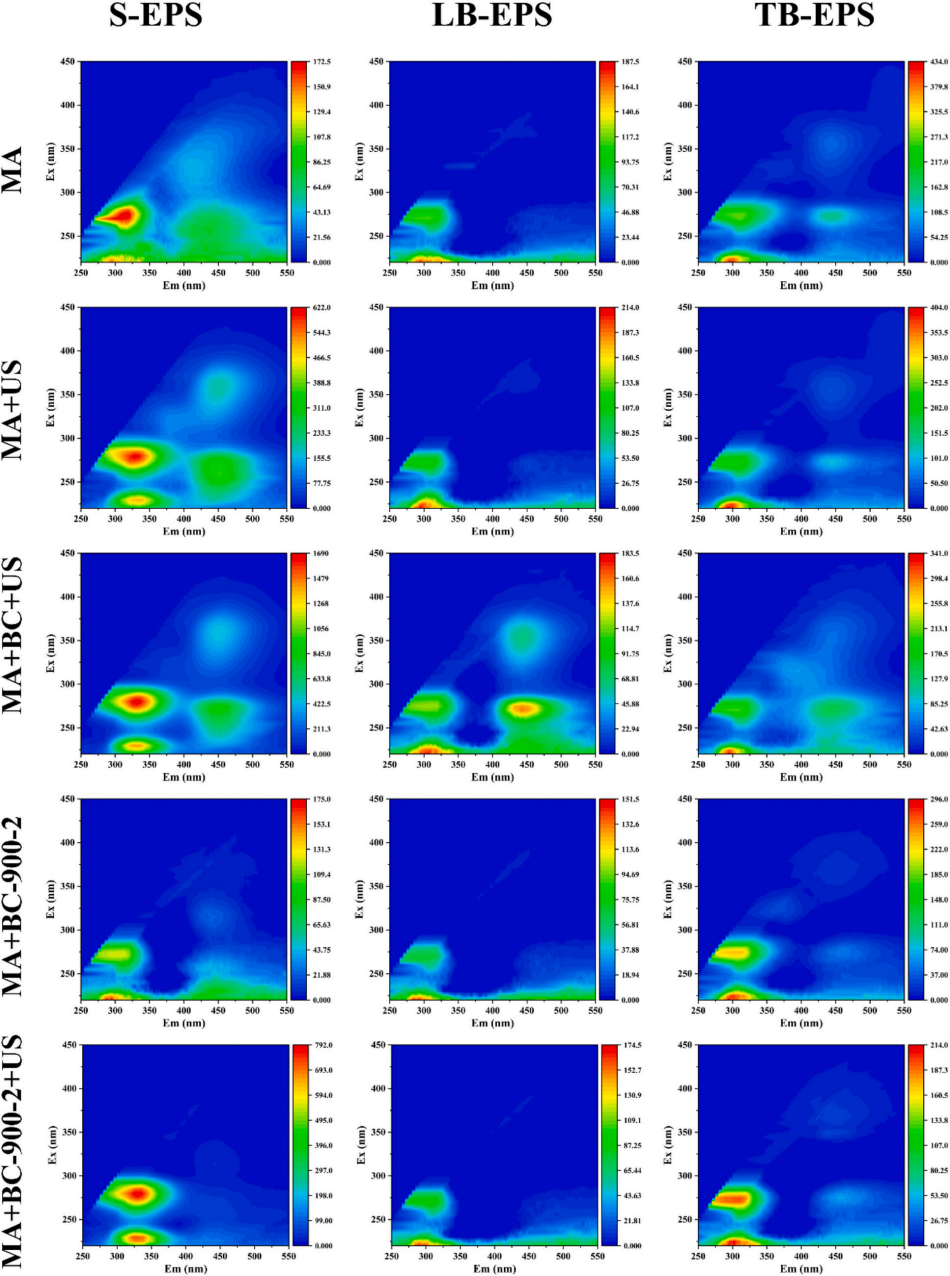
Fluorescence characteristics of soluble/colloidal organic matter in treated algal suspensions. Three-dimensional excitation-emission matrix (3D-EEM) fluorescence spectra of the filtered supernatant (retention on 0.45 μm membrane) from different treatment systems. Spectra represent the soluble and colloidal fraction (including S-EPS, LB-EPS, and TB-EPS) of algal-derived organic matter, excluding cellular debris.

Three-dimensional excitation–emission matrix (3D-EEM) fluorescence spectroscopy was used to characterize the fluorescence features of dissolved AOM (Fig. 4). The dissolved organic matter in the BC-900-2/US system showed prominent protein-like fluorescence peaks (Ex/Em = 220–250/300–380 nm, corresponding to tyrosine-like and tryptophan-like substances), whereas the intensity of humus-like fluorescence peaks was relatively low. These fluorescence characteristics indicate enrichment of protein-like fluorophores and a relatively low contribution of humus-like fluorescence [59], [60]. However, 3D-EEM fluorescence characteristics alone do not directly establish the molecular weight or bioavailability of the released organic matter.

**Fig. 5:**
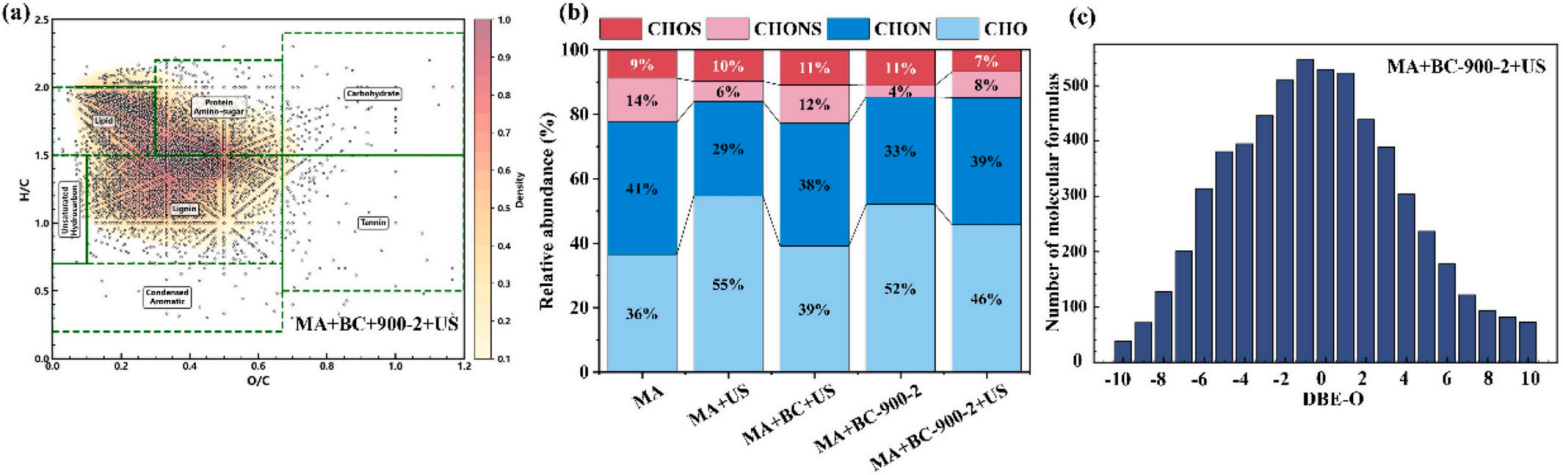
Molecular composition of algal-derived organic matter characterized by FT-ICR-MS (a) Van Krevelen diagrams of algal-derived dissolved organic matter in BC-900-2/ultrasound treatment group, the regions are divided into lipids, protein/amino sugars, lignin-like, carbohydrates, tannins and condensed aromatics. (b) Relative abundance of molecular classes (CHO, CHON, CHONS, CHOS) in algal-derived organic matter detected by FT-ICR-MS. (c) DBE-O distribution of algal-derived organic molecules in the MA + BC-900-2 + US.

Fourier transform ion cyclotron resonance mass spectrometry (FT-ICR-MS) was further used to resolve the molecular composition of AOM at the molecular level. As shown in Fig. 5, CHON molecules accounted for 39% of the total detected molecules in the BC-900-2/US system, followed by CHO molecules (46%), CHONS (8%) and CHOS (7%), indicating a high relative abundance of nitrogen-containing organic components [61]. Van Krevelen diagram analysis showed that the organic molecules were mainly distributed in the protein/amino sugar region, lignin-like region and lipid region, with no obvious accumulation in the tannin or condensed aromatic regions (Fig. 5a, Fig. S10.) [62]. The double bond equivalent minus oxygen (DBE-O) values were mainly concentrated in the range of − 1 to 0 (Fig. 5c), indicating moderate oxidation and unsaturation without obvious accumulation of highly condensed refractory components. These compositional characteristics have previously been associated with potentially bioavailable organic matter [63]. However, molecular-formula distributions alone do not directly demonstrate bioavailability. Although FT-ICR-MS analysis showed no obvious accumulation of highly condensed aromatic molecular formulas (e.g., PAHs) or halogenated compounds, formula-level FT-ICR-MS data alone cannot identify all individual compounds or exclude the presence of potentially toxic species.

**Fig. 6:**
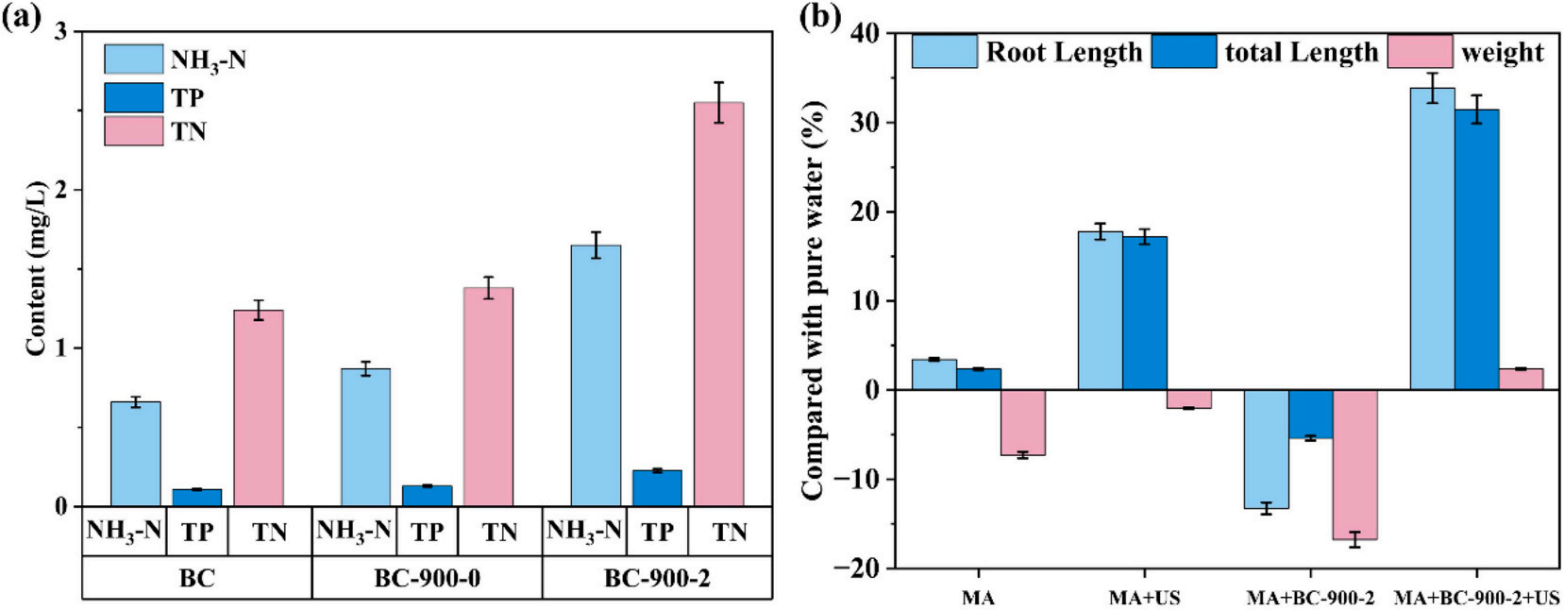
Agricultural valorization potential of the sonodynamic treated algal product. (a) NH_3_-N, TN and TP release concentrations of different biochar materials after 24 h water leaching. (b) Quantitative statistics of root length, total length and fresh weight of soybean sprouts, data are presented as percentage change relative to the pure water control. All data are presented as mean ± s.d. of three independent biological replicates.

Leaching experiments were carried out to evaluate the nutrient release capacity of biochar-algae composite precipitates (Fig. 6a). BC-900-2 showed the highest nitrogen and phosphorus release capacity, with NH_3_-N, total nitrogen (TN) and total phosphorus (TP) release concentrations of 1.64 mg/L, 2.54 mg/L and 0.23 mg/L after 24 h water immersion, which were 2.48 times, 2.05 times and 2.56 times that of raw BC, respectively. Pure water was used as the extractant in this study, which conforms to the standard laboratory method for detecting the maximum nutrient release potential of organic fertilizers, and the nutrient release rule in actual farmland soil environment will be verified in subsequent pilot studies [33].

The soybean germination test is a widely accepted rapid screening method for assessing the phytotoxicity of organic fertilizers. Pot experiments with soybean sprouts were conducted to verify the actual plant growth-promoting effect of the treated products (Fig. 6b-c). Soybean sprout is an internationally recognized model organism for preliminary screening of plant toxicity of agricultural organic materials, and its 7-day growth index can preliminarily reflect the biological safety and application potential of the product. The results showed that different treatment systems had distinct effects on sprout growth. The raw algae group only showed slight promotion in root length (+3%) and total length (+2%), but reduced fresh weight by 7% compared with the pure water control. Single US treatment improved the growth promotion effect, with root length increased by 18% and total length increased by 17%, while fresh weight only decreased by 2%. The BC-900-2 settlement group without ultrasound showed obvious inhibition effect, with root length, total length and fresh weight decreased by 13%, 5% and 17% respectively, which might be due to the adsorption and fixation of bioavailable components by biochar. Notably, the BC-900-2/US system showed the most significant growth-promoting effect. Root length, total length and fresh weight increased by 34%, 31% and 2% respectively compared with the pure water control, preliminarily confirming the feasibility of transforming cyanobacteria pollutants into plant growth-promoting resources. Multi-species whole growth period agricultural verification should be carried out in subsequent studies.

### 3.5 Life cycle analysis

**Fig. 7:**
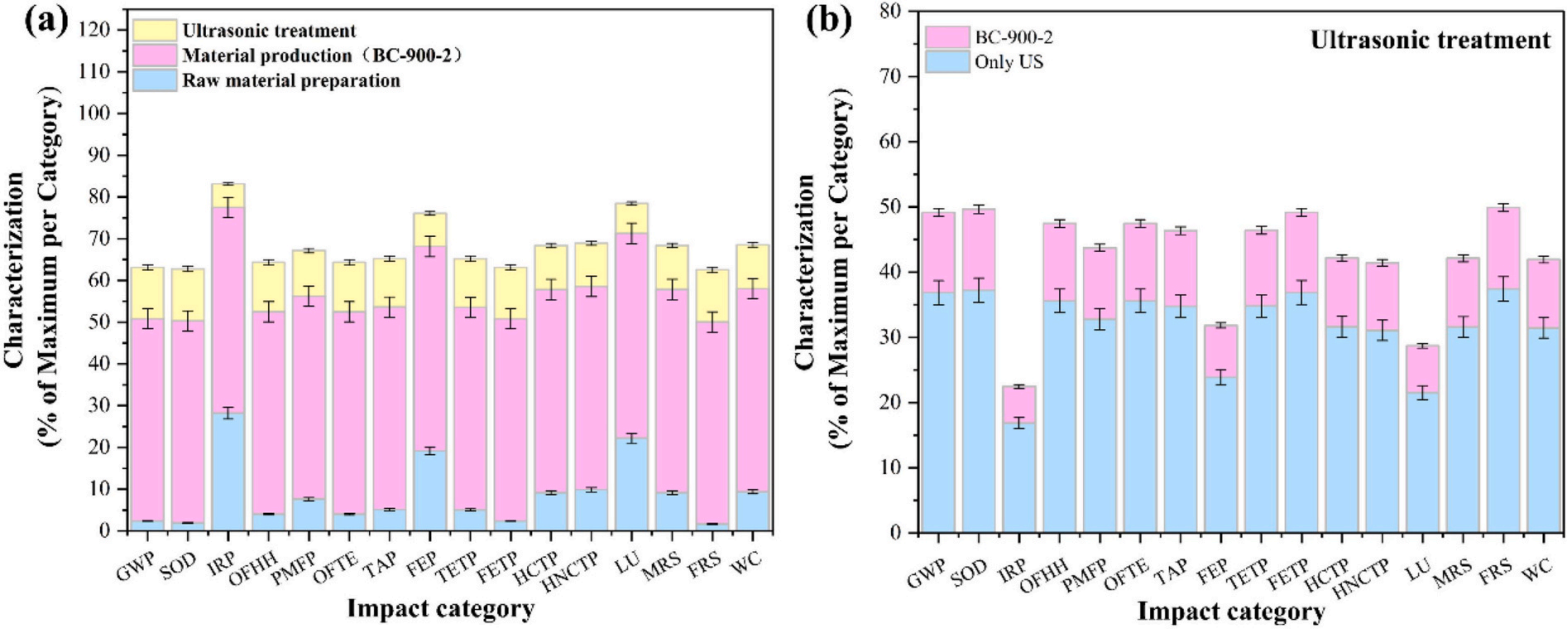
Life cycle environmental impact assessment of the BC-900-2/ultrasonic system for cyanobacterial removal: (a) Contribution of raw material preparation, material production (BC-900-2), and ultrasonic treatment across 16 impact categories. (b) Comparison of environmental impacts between BC-900-2-assisted and sole ultrasonic (Only US) treatment. All results are expressed as normalized percentage contributions.

This study follows ISO 14040/44 standards to carry out life cycle assessment. The functional unit is defined as treatment of 1 m^3^ cyanobacteria-laden water with algae removal efficiency reaching 90%. Background data is sourced from the Ecoinvent 3.9 database combined with China's localized agricultural production dataset. The system boundary covers raw material acquisition, biochar production, ultrasonic treatment and catalyst recovery. Fig. 7a presents the distribution of life cycle contributions for the BC-900-2 coupled ultrasonic system across 16 environmental impact categories under single use scenario. A consistent pattern is observed. The primary environmental burden is concentrated in the material production stage. Its contribution remains stable at approximately 48–49% for most impact categories, which is significantly higher than that of the raw material preparation stage around 2–28% and the ultrasonic treatment stage around 5–12%. Specifically, the raw material preparation stage accounts for relatively higher shares in certain categories, such as ionizing radiation IRP around 28%, freshwater eutrophication FEP around 19%, and land use LU around 22%. These differences are mainly driven by the fertilizer input during bamboo planting and long-distance transportation emission of raw materials. In contrast, the ultrasonic treatment stage maintains a contribution of about 10–12% in categories including global warming potential GWP around 12.29%, ozone formation, and fossil resource consumption. These results indicate that the dominant environmental impact of the current system under single use scenario originates from the high-temperature pyrolysis and iron-loading processes involved in material production [64]. Fig. 7b provides a comparative analysis of the ultrasonic treatment subsystem under the same 90% algae removal efficiency requirement, contrasting the “Only US” scenario with the “BC-900-2 coupled with Ultrasound BC-900-2 + US” scenario. The results show that incorporating the material significantly reduces the environmental burden of the ultrasonic stage across all impact categories, with reductions generally exceeding 60%. For example, the contribution to GWP decreased from 36.87% to 12.29%, to stratospheric ozone depletion SOD from 37.24% to 12.41%, to fine particulate matter formation PMFP from 32.81% to 10.94%, to freshwater eutrophication FEP from 23.88% to 7.96%, and to water consumption WC from 31.46% to 10.49%. This pronounced reduction directly reflects the enhanced sonochemical efficiency due to the addition of BC-900-2, which shortens the required treatment time from 45 min to 10 min under the same efficiency target and lowers the electrical energy consumption per unit of treatment. In other words, BC-900-2 not only acts as a catalytic medium but also intensifies the cavitation effect, leading to superior energy efficiency during system operation. Therefore, the comparison with the sole ultrasound process highlights the energy-efficiency benefit of catalytic intensification, while future work should further include full-chain benchmarking against conventional algal removal technologies.

The developed system constructs a synergistic logic of “low material input − process intensification − net energy consumption reduction”. Although the material production stage brings a certain initial environmental burden under the single-use scenario, the life cycle assessment results show its environmental friendliness [65]. In addition to pollutant control, the system breaks through the traditional end-of-pipe treatment logic. The algal degradation products were enriched in protein/amino sugar-like, lipid-like, and lignin-like components with moderate oxidation states, exhibiting molecular signatures often associated with bioavailable organic matter. Pot experiments showed that these products increased soybean sprout root length and total length by 34% and 31%, respectively, indicating preliminary feasibility of converting cyanobacterial waste into plant growth-promoting resources. This integrated paradigm balancing treatment efficiency and resource recovery fully demonstrates the technical value of the system for cyanobacterial bloom management.

Cyanobacterial bloom control has long been trapped in the trade-off among treatment efficiency, operational cost and secondary pollution risk, and the lack of sustainable algal waste disposal pathways has become a major bottleneck restricting the large-scale application of existing technologies. This work addresses this long-standing challenge by developing a low-cost Fe/KOH co-modified bamboo biochar sonosensitizer, which realizes the dual goals of high-efficiency cyanobacteria elimination and algal waste valorization in a single system. Compared with widely reported sonosensitizers including TiO_2_, noble metal-based nanomaterials and metal–organic framework derivatives, the biochar-based material developed in this study has prominent cost advantages. Bamboo as raw material is widely distributed in global subtropical and temperate regions with an annual output of more than 30 million tons worldwide, and the one-step pyrolysis modification process is simple to operate, with a production cost of only 1/8 ∼ 1/20 of commercial TiO_2_ sonosensitizers, fully meeting the requirements of large-scale preparation and practical engineering application. The triple synergetic mechanism of “cavitation enhancement-interfacial electron conduction-Fenton-like cycle” proposed in this study clarifies the multi-scale structure–activity relationship of biochar-based sonosensitizers for cyanobacteria removal, which provides a reference for the design of high-performance, low-cost carbon-based sonocatalytic materials for water treatment scenarios.

Unlike most previous sonodynamic algae removal studies that focused primarily on pollutant removal efficiency, this study systematically characterized the molecular composition of algal transformation products using high-resolution FT-ICR-MS. The results showed the presence of protein/amino sugar-like, lipid-like, and lignin-like molecular fractions, together with no obvious accumulation of highly condensed aromatic molecular formulas. These compositional characteristics, combined with the growth-promoting effects observed in the soybean sprout assay, suggest that the treated products retained potentially bioavailable organic fractions and possessed preliminary resource-utilization potential. However, the present molecular characterization and plant-growth assay do not demonstrate that the products were dominated by bioavailable components or exclude the presence of all potentially toxic transformation intermediates. Therefore, the environmental safety and bioavailability of the treated products require further systematic evaluation. The integration of water pollution control and agricultural resource utilization aligns with UN Sustainable Development Goal 6 (clean water and sanitation) and Goal 2 (zero hunger) and may contribute to the development of a circular framework linking eutrophic-water treatment, algal-waste utilization, and agricultural production.

It should be noted that the current study was conducted at the laboratory scale. Complex constituents in actual eutrophic waters, such as humic substances and suspended particles, may compete for active sites and consume reactive oxygen species, thereby reducing algal removal efficiency. Future research will therefore focus on three directions. First, pilot-scale tests will be conducted using actual eutrophic waters to systematically investigate the effects of complex water matrices on treatment performance and to establish operating-parameter regulation strategies for practical applications. Second, the material preparation process will be optimized by adjusting the pyrolysis atmosphere and Fe-loading method to reduce production costs and improve material stability and reusability. Third, targeted identification of transformation intermediates, direct bioavailability measurements, metal-residue analysis, and multi-species ecotoxicity assessments will be conducted to further evaluate the environmental safety and resource-utilization potential of the treated products.

## 4 Conclusion

This study developed a series of low-cost iron‑potassium co‑modified bamboo‑derived biochar materials as high-performance sonosensitizers for sonodynamic algae removal. The optimal candidate BC‑900-2 is prepared at a pyrolysis temperature of 900 °C with 2 mol/L Fe_2_(SO_4_)_3_ loading. Under the combined treatment of 10-min 40 kHz ultrasound and 30-min settlement, BC‑900‑2 achieved more than 90% removal of *Microcystis aeruginosa* at an initial concentration of 2 × 10^6^ cells/mL at a dosage of 400 mg/L. Under the tested laboratory conditions, no detectable increase in dissolved microcystin-LR was observed following treatment. However, this finding should not be generalized to higher cyanobacterial densities, other microcystin congeners, or natural water matrices without further validation. The high sonocatalytic activity of BC-900-2 originates from four synergistic structural advantages. These include a developed mesoporous structure, uniformly dispersed Fe_3_O_4_ active sites, stable Fe-O-C interfacial bonding, and a locally graphitized carbon skeleton. A triple synergetic sonodynamic mechanism is confirmed. The porous structure enhances cavitation to promote reactive oxygen species generation. The graphitized carbon skeleton accelerates interfacial electron transfer. The Fe active sites sustain a Fe^2+^/Fe^3+^ cycle to continuously produce •OH, which acts as the dominant reactive oxygen species responsible for oxidative damage and lysis of algal cells. Unlike conventional technologies that focus solely on pollutant removal, this work further demonstrates that the algal degradation products from the BC-900-2/ultrasound system are characterized by molecular signatures resembling protein/amino sugar, lipid, and lignin-like components with a moderate oxidation degree, suggesting the presence of potentially bioavailable fractions. Pot experiments confirmed that these products significantly promoted soybean sprout growth, increasing root length and total length by 34% and 31%, respectively, compared with the pure water control, demonstrating the potential of converting cyanobacterial waste into plant-growth-promoting resources. Life cycle assessment results show that the global warming potential of the system is 0.09 kg CO_2_ eq per m^3^ of treated water, verifying its environmental friendliness. This study introduces a promising technical paradigm that integrates eutrophic water treatment with circular agricultural utilization, offering a potential solution to the long-standing challenge of algal waste disposal in traditional cyanobacterial management, and aligning with the objectives of sustainable development goals related to clean water supply and waste valorization. The current study is still at the laboratory small-scale stage, and the treatment performance in complex actual water bodies needs further pilot validation. Future work will focus on pilot‑scale validation in real water bodies and process optimization to facilitate large‑scale application of this technology.

## CRediT authorship contribution statement

**Yunxiao Zhu:** Writing – original draft, Data curation. **Fulong Chen:** Writing – original draft, Data curation. **Xiaoqing Qian:** Investigation. **Juanjuan Wang:** Investigation, Conceptualization. **Xiaoge Wu:** Writing – review & editing, Supervision, Investigation, Data curation, Conceptualization. **Juan Tu:** Writing – review & editing, Investigation, Funding acquisition. **Jun Wu:** Writing – review & editing, Investigation, Funding acquisition.

## Declaration of competing interest

The authors declare that they have no known competing financial interests or personal relationships that could have appeared to influence the work reported in this paper.
