# Supplementary material for: Designing an eco-efficient sonocatalytic process: iron-modified bamboo biochar for cyanobacteria elimination and algal organic matter valorization potential
Source: Ultrason Sonochem. 2026 Aug 1;132:107988. doi: 10.1016/j.ultsonch.2026.107988 (PMC13474645; doi:10.1016/j.ultsonch.2026.107988)
Supplement: Supplementary file 1 [file mmc1.docx]

Supplementary material

Designing an Eco-Efficient Sonocatalytic Process: Iron-Modified Bamboo Biochar for Cyanobacteria Elimination and Algal Organic Matter Valorization Potential

Yunxiao Zhu^a^, Fulong Chen^a^, Xiaoqing Qiana, Juanjuan Wang^a,b^, Xiaoge Wu^a,b*^, Juan Tu^c*^, Jun Wu^d*^.

^a^ College of Environmental Science and Engineering, Yangzhou University, Yangzhou 225009, China

^b^ Key Laboratory of Cultivated Land Quality Monitoring and Evaluation, Ministry of Agriculture and Rural Affairs, Yangzhou 225009, China

^c^ Key Laboratory of Modern Acoustics, Department of Physics, Collaborative Innovation Center of Advanced Microstructure, Nanjing University, Nanjing, China

Corresponding authors.

[xgwu@yzu.edu.cn](mailto:xgwu@yzu.edu.cn); juantu@nju.edu.cn ;

# S1.0 Figures

**Fig. S1.** Effects of •OH quencher (tert-butanol; TBA) concentration on algae removal efficiency in the (a) US, (b) BC, and (c) BC-900-0 systems.

**Fig. S2.** Pore size distribution of BC, BC-900-0 and BC-900-2 obtained from Brunauer-Emmett-Teller (BET) test.

**Fig. S3.** X-ray photoelectron spectroscopy (XPS) Fe 2p high-resolution spectrum of BC-900-2 after sonodynamic treatment.

**Fig. S4.** Luminol-enhanced sonochemiluminescence images of different reaction systems.

**Fig. S5.** Effects of temperature, pH, and biochar dosage on KI-derived equivalent H₂O₂ production in different systems. [1-3]

**Fig. S6.** (a) Time-dependent UV–vis absorption spectra of 1,3-diphenylisobenzofuran (DPBF) during treatment. (b) Time-dependent UV–vis absorption spectra of methylene blue (MB) during treatment. (c) UV–vis diffuse reflectance spectra and band gap fitting plots; (d) valence band XPS spectrum of BC; (e) valence band XPS spectrum of BC-900-2; (f) valence band XPS spectrum of BC-900-0; (g) schematic band positions of different samples All data are presented as mean ± s. d. of three independent replicates.

**Fig. S7. Flow cytometry analysis of *Microcystis aeruginosa* under different treatments.** (a) Viability assessment using PI/SYTO 9 double-staining. Dot plots distinguish four populations: viable cells (SYTO 9+/PI-, bottom right), dead or double-stained cells (SYTO 9+/PI+, top right), dead cells (SYTO 9-/PI+, top left), and unstained cells (SYTO 9-/PI-, bottom left). (b) Intracellular reactive oxygen species (ROS) levels detected by flow cytometry. (c) Esterase activity (as an indicator of metabolic activity) assessed by fluorescein diacetate (FDA) staining and flow cytometry.

**Fig. S8.** Fourier transform infrared (FTIR) spectra of biochar, Microcystis aeruginosa (MA), and their composite under different treatment conditions.

**Fig. S9.** Analytical results of dissolved organic carbon (DOC), UV absorbance, and extracellular polymeric substances (EPS) in Microcystis aeruginosa cultures under different treatment systems.

**Fig. S10.** Van Krevelen diagrams of algal-derived dissolved organic matter under different treatment conditions: (a) MA, (b) MA+US, and (c) MA+BC-900-2. The molecular formulas were classified according to their H/C and O/C ratios into lipids, protein/amino sugars, lignin-like compounds, carbohydrates, tannins, and condensed aromatics. The color scale represents the molecular density distribution.

**Fig. S11.** DBE-O distribution of algal-derived organic molecules in different treatment systems. (a) MA; (b) MA + US; (c) MA + BC + US; (d) MA + BC-900-2 + US.

.

# S2.0 Tables

Table S1. Comparison of different enhanced algae removal technologies with this study

| **Physical field** | **Algal species** | **Initial concentration** | **Treatment conditions** | **Catalyst** | **Removal efficiency** | **Reactive species** | **Mechanism** | **Reference** |
| --- | --- | --- | --- | --- | --- | --- | --- | --- |
| Ultrasound | *Microcystis aeruginosa* | 1.3×10^7^ cells/mL | 90 s | TiO_2_/biochar | 92% | •OH（dominant）、•O_2_^−^ | ROS-induced membrane damage enhances coagulation–sedimentation | [4] |
| Ultrasound | *Microcystis* *aeruginosa* | 0.7×10⁷ cells/mL | US pretreatment + coagulation | PAC | >90% | •OH | Ultrasound reduces stability and promotes coagulation–sedimentation (oxidation, mechanical disruption) | [5] |
| Light | *Microcystis aeruginosa* | 2.7×10^6^ cells/mL | Visible light, 6 h | g-C_3_N_4_/EP-Al_2_O_3_ | 74.4% | •OH（dominant）、•O_2_^−^ | Photocatalytic ROS-induced cell damage | [6] |
| Magnetic | *Chlamydomonas sp.* | 1.5×10^7^ cells/mL | Rapid/slow mixing + magnetic settling (15/25/5–10 min) | Fe_3_O_4_/CPAM | >97% | None | Charge neutralization and bridging; magnetic-enhanced flocculation | [7] |
| Electric | *Microcystis aeruginosa* | Not reported | 30 min | Al electrodes (anode) and graphite cathode | 98% | None | Electrocoagulation and flotation | [8] |

Table S1. Comparison of different enhanced algae removal technologies with this study (continued)

| **Physical field** | **Algal species** | **Initial concentration** | **Treatment conditions** | **Catalyst** | **Removal efficiency** | **Reactive species** | **Mechanism** | **Reference** |
| --- | --- | --- | --- | --- | --- | --- | --- | --- |
| Gravity | *Microcystis aeruginosa* | 2×10^6^ cells/mL | 1 min rapid mixing + 20 min slow mixing + 30 min settling | AlCl_3_ | ~100% | None | Coagulation–sedimentation removal | [9] |
| **Ultrasound** | ***Microcystis aeruginosa*** | **2×10^6^ cells/mL** | **US 10 min + settling 30 min** | **BC-900-2** | **>90%** | **•OH（dominant）** | **ROS-induced cell damage & interfacial reactions** | **This work** |

**Table S2**. Preparation conditions and nomenclature of the bamboo-derived biochar samples

| **Sample** | **Fe₂(SO₄)₃ concentration (mol L⁻¹)** | **Pyrolysis temperature (°C)** |
| --- | --- | --- |
| BC | **—** | **—** |
| BC-400-0 | 0 | 400 |
| BC-400-1 | 1 | 400 |
| BC-400-2 | 2 | 400 |
| BC-400-4 | 4 | 400 |
| BC-900-0 | 0 | 900 |
| BC-900-1 | 1 | 900 |
| BC-900-2 | 2 | 900 |
| BC-900-4 | 4 | 900 |

# S3.0 Text S1

To quantitatively evaluate the cooperative enhancement between BC-900-2 and ultrasound in algal organic matter release/transformation, a UV₂₅₄-based synergy index was calculated. [10, 11]The calculation was adapted from the commonly used synergy-index approach in combined ultrasound-based advanced oxidation processes, in which the observed response of the combined process is compared with the expected additive response of the individual processes.

UV₂₅₄ was selected as the response indicator because it reflects UV-absorbing/chromophoric dissolved organic matter in water：

$$A_{\mathrm{expected}}=A_{US}+A_{BC}-A_{MA}$$

$$SI_{\mathrm{UV}254}=\frac{A_{\mathrm{BC}+\mathrm{US}}}{A_{\mathrm{expected}}}$$

where $A_{\mathrm{MA}}$, $A_{\mathrm{US}}$, $A_{\mathrm{BC}}$, and $A_{\mathrm{BC}+\mathrm{US}}$represent the UV₂₅₄ absorbance values of the untreated algal suspension, ultrasound-only treatment, BC-900-2-only treatment, and combined BC-900-2/ultrasound treatment, respectively. $SI_{\mathrm{UV}254}>1$ indicates that the observed UV₂₅₄ response of the combined treatment exceeds the expected additive response of ultrasound alone and BC-900-2 alone, suggesting positive synergistic enhancement in UV₂₅₄-active algal organic matter release/transformation.

The calculated $SI_{\mathrm{UV}254}$was 1.82, indicating that the combined BC-900-2/US treatment produced a stronger UV₂₅₄ response than the expected additive response of ultrasound alone and BC-900-2 alone.

# References

[1] K.S. Suslick, Sonochemistry, Science 247 (1990) 1439-1445. <https://doi.org/10.1126/science.247.4949.1439>

[2] M. Cheng, C. Lai, Y. Liu, G. Zeng, D. Huang, C. Zhang, L. Qin, L. Hu, C. Zhou, W. Xiong, Metal-organic frameworks for highly efficient heterogeneous Fenton-like catalysis, Coordination Chemistry Reviews 368 (2018) 80-92. <https://doi.org/10.1016/j.ccr.2018.04.012>

[3] P.R. Gogate, A.B. Pandit, A review and assessment of hydrodynamic cavitation as a technology for the future, Ultrason. Sonochem. 12 (2005) 21-27. <https://doi.org/10.1016/j.ultsonch.2004.03.007>

[4] J. Wang, W. Li, X. Wu, Microcystis aeruginosa removal by the combination of ultrasound and TiO_2_/biochar, RSC Advances 11 (2021) 24985-24990. <https://doi.org/10.1039/d1ra03308e>

[5] Y. Huang, L. Li, X. Luan, X. Wei, H. Li, N. Gao, J. Yao, Ultrasound-enhanced coagulation for cyanobacterial removal: Effects of ultrasound frequency and energy density on coagulation performance, leakage of intracellular organic matters and toxicity, Water Res. 201 (2021). <https://doi.org/10.1016/j.watres.2021.117348>

[6] J. Song, X. Wang, J. Ma, X. Wang, J. Wang, J. Zhao, Visible-light-driven in situ inactivation of Microcystis aeruginosa with the use of floating g-C3N4 heterojunction photocatalyst: Performance, mechanisms and implications, Appl. Catal. B 226 (2018) 83-92. <https://doi.org/10.1016/j.apcatb.2017.12.034>

[7] L. Chen, X. Wu, C. Liu, Y. Zhao, Q. Huang, Study on removal of Microcystis aeruginosa and Cr (VI) using attapulgite-Fe3O4 magnetic composite material (MCM), Algal Research 60 (2021) 102501. <https://doi.org/10.1016/j.algal.2021.102501>

[8] T.A. Meetiyagoda, T. Fujino, Comparison of Different Anode Materials to Remove Microcystis aeruginosa Cells Using Electro-Coagulation–Flotation Process at Low Current Inputs, Water 12 (2020) 3528. <https://doi.org/10.3390/w12123528>

[9] F. Sun, H.-Y. Pei, W.-R. Hu, C.-X. Ma, The lysis of Microcystis aeruginosa in AlCl3 coagulation and sedimentation processes, Chem. Eng. J. 193-194 (2012) 196-202. <https://doi.org/10.1016/j.cej.2012.04.043>

[10] M. Philibert, S. Luo, L. Moussanas, Q. Yuan, E. Filloux, F. Zraick, K.R. Murphy, Drinking water aromaticity and treatability is predicted by dissolved organic matter fluorescence, Water Res. 220 (2022). <https://doi.org/10.1016/j.watres.2022.118592>

[11] O. Hamdaoui, S. Merouani, M. Ait Idir, H.C. Benmahmoud, A. Dehane, A. Alghyamah, Ultrasound/chlorine sono-hybrid-advanced oxidation process: Impact of dissolved organic matter and mineral constituents, Ultrason. Sonochem. 83 (2022). <https://doi.org/10.1016/j.ultsonch.2022.105918>
